# A Post-pleistocene Calibrated Mutation Rate from Insect Museum Specimens

**DOI:** 10.1371/currents.tol.aba557de56be881793261f7e1565cf35

**Published:** 2018-07-13

**Authors:** Gideon Ney, Katy Frederick, Johannes Schul

**Affiliations:** Department of Biological Sciences, University of Missouri, Columbia, Missouri, United States; Department of Biological Sciences, University of Missouri, Columbia, Missouri, United States; Department of Biological Sciences, University of Missouri, Columbia, Missouri, United States

## Abstract

Quantifying the age of recent species divergence events can be challenging in the absence of calibration points within many groups. The katydid species *Neoconocephalus lyristes* provides the opportunity to calibrate a post-Pleistocene, taxa specific mutation rate using a known biogeographic event, the Mohawk-Hudson Divide. DNA was extracted from pinned museum specimens of *N. lyristes* from both Midwest and Atlantic populations and the mitochondrial gene COI sequenced using primers designed from extant specimens. Coalescent analyses using both strict and relaxed molecular clock models were performed in BEAST v1.8.2. The assumption of a strict molecular clock could not be rejected in favor of the relaxed clock model as the distribution of the standard deviation of the clock rate strongly abutted zero. The strict molecular clock model resulted in an intraspecific calculated mutation rate of 14.4-17.3 %/myr, a rate substantially higher than the common rates of sequence evolution observed for insect mitochondrial DNA sequences. The rate, however, aligns closely with mutation rates estimated from other taxa with similarly recent lineage divergence times.

## Introduction

In recent years, many examples of rapid speciation and diversification occurring during the last glacial cycle (i.e., within 500 kyr BP 1), or even after the last glacial maximum (LGM, 19 kyr BP 2) have been described. Arguably, the most impressive examples of rapid diversification are the cichlid radiation events within the African Rift Valley, where a small number of founding species diversified into hundreds of species after the LGM 3^,^^4^^,^^5^. Other examples include the old world pea aphids 6, North American songbirds 7^,^^8^^,^^9^, and the threespine sticklebacks of British Columbia 10; in some cases, significant diversification arose in as little as 50 years 11.

The accurate timing of diversification events allows us to better understand the mechanisms leading to phenotypic diversification and/or speciation. Molecular clock techniques allow the timing of diversification events based on estimates of the rate of genetic mutations per unit time 12^,^^13^. Mutation rates are gene specific and can vary between lineages and through time within a lineage 14^,^^15^. Therefore, accurate dating using a molecular clock requires reliable calibration of the rate of sequence evolution for that particular group, time interval, and gene. Rates can be calibrated using nodes dated from fossils 16 and from biogeographic vicariance events 17^,^^18^.

Estimates of nucleotide evolution vary greatly dependent with the age of the calibrating point, with younger calibration points resulting in higher rate estimates 19^,^^20^^,^^21^. Fossils and most biogeographic events are ancient (millions of years old) and are appropriate for the dating of similarly ancient events. The few available rate estimates using very young age calibration points (<200kyrs 22^,^^23^^,^^24^) suggest an exponential increase of estimated rates 25^,^^26^^,^^27^; additional data is needed to support this pattern. The exponential pattern of estimates is likely an artifact of the estimation methods and does not reflect true differences in rates on nucleotide evolution 19. One reason for the small number of estimates for recent lineage divergences is that suitably recent calibration points are scarce 25, since these events are too recent to use fossil evidence.

Here we use a postglacial vicariance event to calibrate a lineage specific mutation rate for North American *Neoconocephalus* katydids. At a time following the LGM, water from the North American Great Lakes drained through the Mohawk-Hudson Outlet to the Atlantic coast 28. Wetland habitats formed within the Hudson and Mohawk Valleys, which allowed coastal plain species to expand their ranges into the wetlands surrounding the Great Lakes 29. The opening of the St. Lawrence Seaway (10,750-10,600 14C yr BP 30), diverted melt water and led to the drying of the wetlands in the Mohawk-Hudson outlet. This vicariance event left disjunct wetland habitats in the Midwest (mainly bogs and fens) and along the Atlantic Coast (bogs and marsh habitat). Such disjunct ranges matching this pattern are found in plant, reptile, amphibian, and insect species possessing a coastal plain affinity 29^,^^31^^,^^32^. *Neoconocephalus lyristes* is an example of such a habitat specialist, limited to bog and fen wetlands. The species’ described range follows the pattern of the Mohawk-Hudson Divide, with isolated populations in the Great Lakes area 31 and North Atlantic Coast (33 Fig. 1).

**Historic collection sites for N. lyristes overlaid with hypothesized range.**
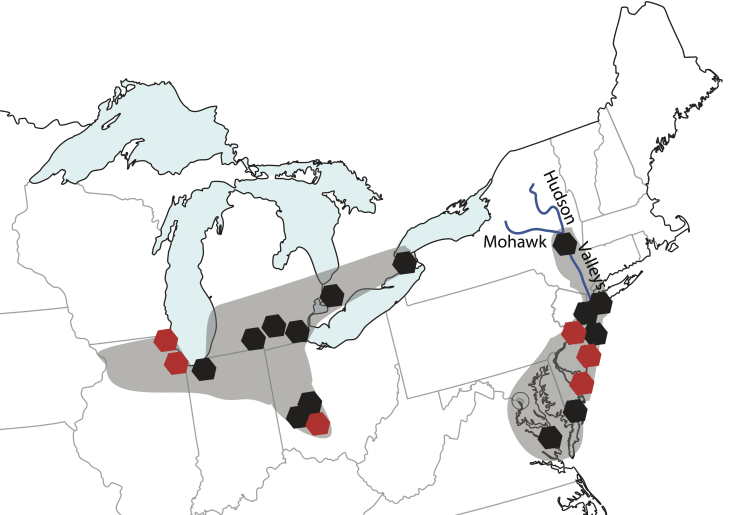
Sites are modified from 34^,^^35^ based on literature and collection records. The collection localities of museum samples used in this study are indicated in red.

The eleven North American *Neoconocephalus* katydid species possess markedly little genetic variation despite their high diversity of species-specific call patterns and may be an example of a recent species radiation 36^,^^37^. The accurate timing of this radiation will help identify the evolutionary mechanisms leading to rapid species diversification observed in this group. *Neoconocephalus lyristes* provides a unique opportunity among species of *Neoconocephalus* for the calibration of a post-Pleistocene mutation rate as gene flow between these two disjunct ranges likely ceased with the draining of the Mohawk-Hudson Outlet (10,750-10,600 14C yr BP 30). Here we sequenced mtDNA from museum specimens representing both populations and estimated an intraspecific mutation rate using a coalescent Bayesian method.

## Methods

Over three years of searching previous collection sites we found only a single extant population of *N. lyristes*, in Cedar Bog Nature Preserve, Urbane, OH, USA. Due to apparent local extinction of *N. lyristes* from most of its Midwest and its entire Atlantic range, we used museum samples collected in the first half of the 20th century. We selected 18 dried *N. lyristes* specimens, from the Hebard Collection at the Academy of Natural Sciences of Drexel University for DNA extraction and analysis. Specimens represent samples from both Atlantic Coastal and Midwest populations (Fig. 1), with collection dates ranging from 1905-1932. We used a non-destructive method for DNA extraction (modified from 38). A hind leg was removed and placed in a 1.5 ml microcentrifuge tube fully submerged in one ml of digestion buffer: 3 mM CaCl2, 2% sodium dodecyl sulphate (SDS), 40 mM dithiothreitol (DTT), 250 mg/ml proteinase K, 100 mM Tris buffer pH 8 and 100 mM NaCl (quantities represent molarity of final concentrations). Hind legs were incubated overnight (17-19 hrs.) at 55°C. Following digestion we removed the hind legs from buffer and placed them in 100% EtOH for two hours to stop enzymatic activity. Extraction of DNA contained in the buffer was completed using the standard Qiagen DNeasy Blood + Tissue Kit (Qiagen Inc., Valencia, CA, USA) extraction method.

Amplification took place in a laboratory without prior exposure to DNA that could be amplified by primers used in this study. Polymerase chain reaction (PCR) prep was performed in a UV hood. All equipment and surfaces were sanitized with a 10% bleach solution and tools were sanitized in a UV Stratalinker 1800. For this study we designed six overlapping primer pairs (Appendix: Supplemental Table 1) around non-variable regions of the mitochondrial gene cytochrome oxidase I (COI). These primers were based upon extant *N. lyristes*,* N. robustus*, and *N. bivocatus* COI sequences and designed using the Primer3 39 plugin in Geneious v6.0.5 40. Each primer pair amplified approximately 150 bp; combined, they provide complete coverage of the 743 bp target region.

PCR amplification was performed on an Eppendorf Mastercycler gradient (Eppendorf-Brinkman Instruments Inc., Westbury, NY, USA) using Taq DNA polymerase (Platinum Taq, Invitrogen Inc., Carlsbad, CA, USA). All primers were used at a concentration of 10 mM. Thermocycling conditions for all six primer-sets are as follows: Hot start at 94°C 2 min, denaturation at 94°C 30 sec, annealing at 56°C 30 sec, extension 72°C 40 sec, repeated 40x, with a final 72°C extension for 7 min. Amplified PCR products were prepared for sequencing using a ExoI/SAP enzymatic cleanup (2.75 μl 10x SAP buffer, 0.5 μl SAP, 0.25 μl ExoI per 20 μl of PCR product) incubated at 37°C for 30 min, followed by 80°C for 15 min to inactivate enzymes. Sequencing was performed at the DNA Core Facility, University of Missouri, Columbia, MO, USA on an ABI 3730 DNA Analyzer, using standard Big Dye Terminator cycle sequencing chemistry (Applied Biosystems, Foster City, CA, USA). Sequences were edited, aligned and trimmed in Geneious v6.0.5 41. We used a global alignment with free end gaps and 70% similarity rule. Regions of sequence with high ambiguity were labeled as missing. One individual, with greater than ten percent ambiguity, was removed from the analysis (m017). Individual m007 failed to amplify. We successfully sequenced COI from 16 individuals.

We evaluated substitution models using jModel Test v0.1.1 42 and found GTR+G to be a suitable model. Phylogenetic analyses were conducted using a coalescent method as implemented in BEAST v1.8.2 xml 43; input files were formatted using BEAUti v1.7.4 43. Our analysis assumed a constant population size for the coalescent inferences 44. We ran this analysis to convergence, performing ten runs with twenty million generations sampled every two thousand trees. We assessed convergence through visual inspection of posterior values among the ten runs in Tracer v1.5 45. This analysis was performed using both a strict 46, as well as a relaxed molecular clock model 13. The Midwest individuals were run both unconstrained as well as constrained to monophyly. The constrained run assured that the age calibration point was assigned to the correct node in all trees 21. To evaluate the influence of the prior settings on the posterior samples, we repeated the analysis as above but without any sequence data.

Using the radiocarbon date of 10,750±150 14C yr BP, the end of 150-300 year period of steady melt water flow following the final large flood through the Hudson Valley at 10,900 14C yr BP 30, we calibrated the calendar age of the Mohawk-Hudson Divide. We performed the radiocarbon to calendar age conversion using the IntCal13 curve in OxCal v4.2 online 47. The age estimate was fixed to the highest likelihood value within the 95% confidence interval; yielding a calibrated date of 10,739.5 cal BP. Being a known biogeographic barrier we allowed the node age prior probability of the Midwest clade to vary along a normal distribution, with the calibrated date as the mean age and a standard deviation of one-thousand years. This allows for the possibility of lineage divergence prior to the biogeographic event, as well as the overestimation of the events age 48. The Euclidean mean and standard deviation priors were set to exponential with mean values of 10 and 0.3 respectively. Convergence of MCMC runs was visualized using Tracer v1.5 45 to ensure that all runs converged. With Tracer v1.5 we ascertained the average mutation rate between populations of *N. lyristes* based on the Mohawk-Hudson calibration. Runs were combined in LogCombiner v1.8.2 49 and a maximum clade credibility consensus tree was formed in TreeAnnotator v1.7.4 50.

## Results

We successfully sequenced 743 bp of the mitochondrial gene COI from sixteen individuals (5 from Midwest and 11 from Atlantic Coast populations, Table 1). Sequence similarity among the 16 samples ranged from 92.0% to 99.8%. We found the greatest diversity within the Atlantic population. The Midwest clade fell within the larger clade of Atlantic Coast *N. lyristes* (Fig. 2). This observation is congruent with the hypothesized biogeographic history of the species where the Midwest populations diverging from the ancestral Atlantic population.

**Table 1: Museum specimen list:** *N. lyristes* pinned specimens obtained from the Hebard collection at the Academy of Natural Sciences of Drexel University. Included is all relevant data from specimen label, as well as the ambiguities present in final sequences. (*) denotes samples removed from analysis for failed amplification or excess ambiguity.

Study reference #	Locality	Collection Date	Collected/ID by	Ambiguities (#/743 bp)
m001	Cape May Court House, NJ	1914	Hebard	0
m002	Cape May Court House, NJ	1914	Hebard	1
m003	Cape May Court House, NJ	1914	Hebard	0
m004	Cape May Court House, NJ	1914	Hebard	1
m005	Cape May Court House, NJ	1914	Hebard	0
m006	Cape May Court House, NJ	1914	Hebard	24
m007	Cedar Swamp, OH	1929	Unknown	N/A*
m008	Cedar Swamp, OH	1932	Edward S. Thomas	25
m009	Cedar Swamp, OH	1932	Edward S. Thomas	0
m010	Chicago, IL (Beach IL)	1906	Unknown	2
m011	Chicago, IL (S. of Jackson Park)	1905	Unknown	18
m012	Chicago, IL (S. of Jackson Park)	1905	Unknown	15
m013	Whitesbog, NJ	1923	Det. D.C. Rentz (1974)	0
m014	Whitesbog, NJ	1923	H. Fox	0
m015	Whitesbog, NJ	1923	Unknown	0
m016	Whitesbog, NJ	1923	H. Fox	0
m017	Whitesbog, NJ	1923	Unknown	103*
m018	Whitesbog, NJ	1923	Unknown	1

**Consensus tree from coalescence analysis using a strict molecular clock model and Midwest clade constrained to monophyly:**
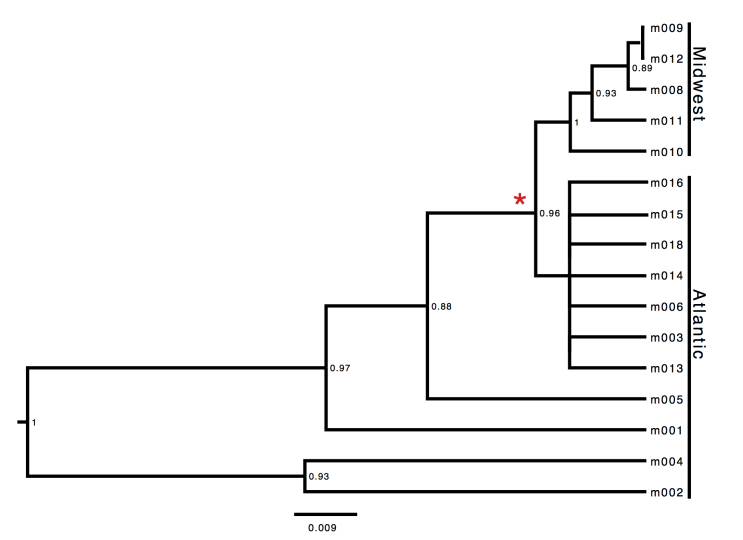
Nodes possessing <0.85 posterior probabilities were collapsed. Red star represents the Mohawk-Hudson Divide, with the prior of the node age set to a normal distribution with a mean age of 10,739.5 cal BP. The Midwest specimen m010 fell outside of the Midwest clade prior to constraining the group to monophyly.

Using the unconstrained coalescence model, four out of five Midwestern individuals formed a clade within the larger clade of Atlantic Coast *N. lyristes.* One Midwest individual (m010) grouped among Atlantic individuals (Appendix: Supplemental Fig. 1). In order to prevent the age calibration point from being assigned to the wrong nodes in some trees we constrained the Midwest clade to monophyly in further analyses 21. The resulting constrained consensus tree (Fig. 2) is congruent with the hypothesized biogeographic history of the species, with the Midwest population diverging from the ancestral Atlantic population.

Using a relaxed clock model, we obtained branch specific mutation rates between 14.4 and 37.5 %/myr from the consensus of the ten runs. The average rate of mutation among branches was 15.8 %/myr, ranging from 15.7-15.9 %/myr between the ten independent runs. The distribution of the standard deviation of the clock rate strongly abutted zero when the relaxed molecular clock was used (Fig. 3). This indicates support for a constant rate of substitution and a strict molecular clock was used 51. The strict molecular clock analysis produced a tree (Fig. 2) with a similar, but not identical, topology to the relaxed clock’s consensus tree. The relationship between Midwest animals and their relationship to the Atlantic clade remained unchanged, with minor changes in the relationships between Atlantic individuals. The strict consensus tree, with Midwest clade constrained to monophyly possessed an average mutation rate of 17.3 %/myr, with mutation rates between the ten runs. Predictably, a slower rate of 14.4 %/myr was obtained when the same analysis was run with individual M010 removed. These two rates, while diverging slightly, both indicate a rate of mutation significantly faster than most reported in the literature 19^,^^20^.

**Distribution of the standard deviation rates from relaxed clock analysis:**
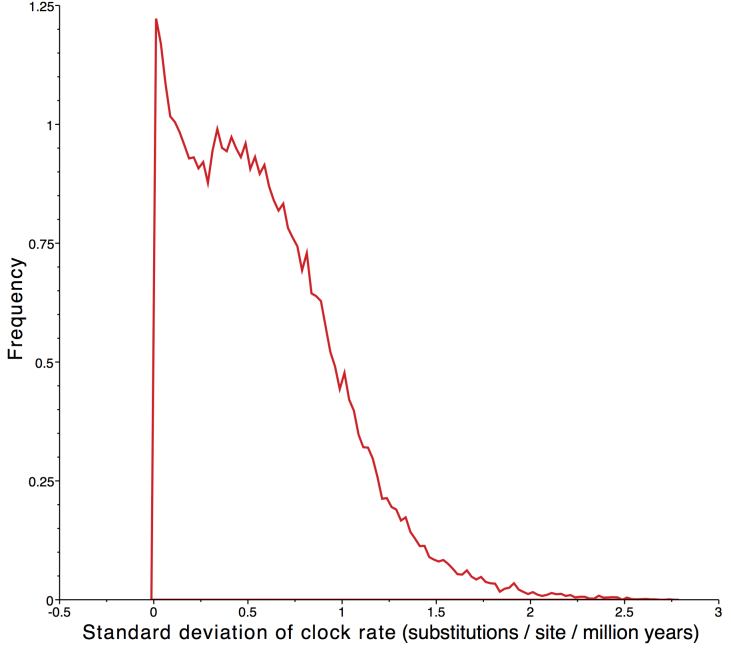
Includes data from ten combined runs (twenty million generations sampled every two thousand trees) using a relaxed molecular clock model. Units for the clock rate are in substitutions per site per million years. The distribution strongly abuts zero, indicating support for a strict molecular clock 51.

## Discussion

Here, we focused on the calibration of an intraspecific mutation rate at a very recent timescale. Evolutionary rates calibrated across divergent timescales can be markedly different 25, with younger calibration dates (<1 Mya) showing substantially higher estimates of rates divergence than older lineages 19^,^^20^. In mammals, for example, the age of the calibration dates shows a negative relationship with estimates for molecular evolutionary rates 19^,^^52^. Metastudies utilizing insect mtDNA rates estimated from both inter- and intraspecific calibrations show a similar pattern to that observed in mammals 20^,^^21^. Available data suggest an exponential increase of estimated rates 22^,^^23^^,^^24^ with decreasing calibration age (Fig. 4). The exponential pattern of estimates is likely an artifact of the estimation methods and does not reflect true differences in rates on nucleotide evolution 19.

**Estimates of evolutionary rates (%/myr) plotted against calibration age (myr):**
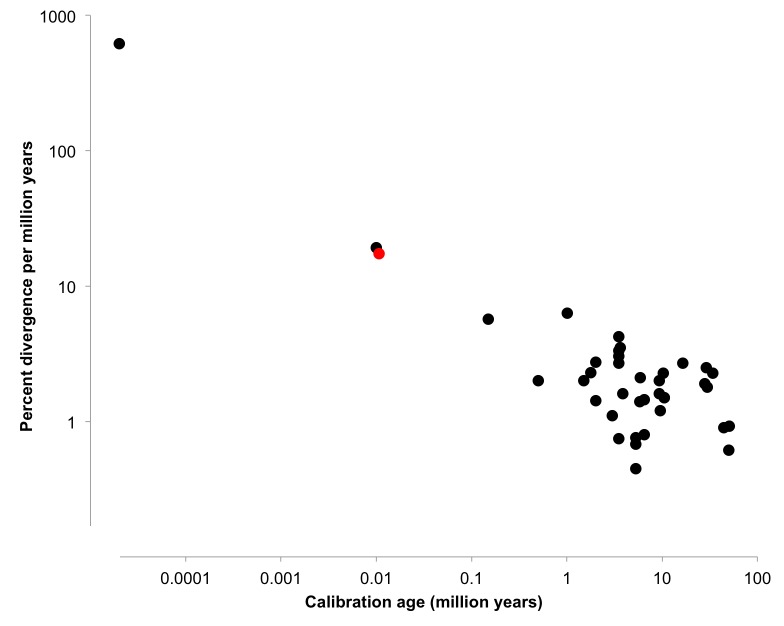
The black data points were obtained directly from 20^,^^21^. The red point represents the mutation rate estimate from this study. Note both axes are in log scale.

The sequence variation among populations has two components, fixed substitutions between them that have accumulated since divergence and current within population variation 19. The fixed substitutions among lineages represent the actual evolutionary divergence. Most of the within population genetic variation will be removed over time by genetic drift and selection and therefore only a small fraction will ultimately contribute to lineage divergence 26^,^^53^. For young divergence times, the within species variation will contribute a much larger fraction of the total nucleotide differences, as only few fixed substitutions have accumulated. For ancient divergence times, in contrast, the same amount of within population variation would be dwarfed by fixed substitutions accumulated since divergence 27^,^^54^. Thus, short calibration times should lead to gross overestimations of evolutionary divergence rates, while ancient calibration times (>1 Mya 54) should provide much more realistic estimates.

**Insect mtDNA rates of mutation:**

We estimated a mutation rate for COI at 14.4-17.3 %/myr, using the strict molecular clock model and a very recent calibration time. Our estimate is significantly higher than the commonly assumed mtDNA mutation rate of 1.15 %/myr 55^,^^56^, which were based on much older divergence times. Estimates of substitution rates calibrated from the age of the Mid-Aegean Trench (9-12 Mya), for example, within an insect model range from 1.0-2.7 %/myr dependent upon application of various substitution and clock models 21. Our estimated rate of 14.4-17.3 %/myr, on the other hand, aligns with estimates found using similarly recent calibration dates (Fig. 4). A mutation rate of 19.2 %/myr was estimated for the European butterfly *Parnassius mnemosyne, *calibrated with a vicariance event at 10,000 years BP 23. Intermediate calibration dates resulted in an intermediate estimate of evolutionary rates. The mutation rate for the North American ground beetle (*Nebria*) was estimated at 5.7 %/myr, using a vicariance event dated to 150,000 years BP 22. Our estimates for *N. lyristes* fit into the exponential pattern previously described (Fig. 4). Thus, this study agrees with the slower estimates of Orthopteran mtDNA sequence evolution and may serve as an internal calibration point for *Neoconocephalus* diversification.

The high mutation rate inferred from our data set could be due to problems in the mathematical models underlying the molecular clock. This seems unlikely, since both fixed clock and relaxed clock models lead to nearly identical results. Furthermore, the close fit of our data point to data from previous work conducted with a variety of methods 20^,^^21^ suggests that our particular methods were not responsible for the high estimated mutation rate.

As more evidence accumulates supporting the occurrence of postglacial species diversification, the greater the need for appropriate tools for timing these events. This will in part include the utilization of young vicariance events for molecular clock calibration. Geologically supported postglacial vicariance events within North America are lacking for many taxa groups 57^,^^58^. The Mohawk-Hudson Divide provides a recent biogeographic vicariance event, with the potential for the calibration of lineage specific mutation rates for a number of plant, amphibian, reptile, and insect groups.

**Use of museum samples:**

The use of ancient DNA (aDNA) samples can be hindered by severe degradation 38^,^^59^. In this study two of the eighteen samples could not be sequenced successfully. These two samples were not the oldest, nor from the same locality. Severe degradation of DNA, beyond that in the other sixteen samples, or a mismatch in primer binding sites may account for failed amplification (Table 1). In those samples that were sequenced successfully ambiguities were high, while this is likely due to the degraded nature of aDNA, the coamplification of nuclear pseudogenes could also lead to such ambiguities. The amplification of relatively short (150 bp) segments increases the likelihood of amplifying pseudogenes, not amplified when targeting longer sequences. Nuclear pseudogenes of COI, while not noted in *Neoconocephalus*, have been found in other Orthopterans 60. We found no internal stop codons within our COI sequences. As internal stop codons are common in pseudogenes, it is unlikely that our data is affected by their presence. Our primers were developed from COI reference sequences from three extant *Neoconocephalus* species. Amplification would therefore not be affected by sequence degradation, as may be the case if primers are developed from the aDNA itself. One concern with the use of aDNA is sequence degradation, with post mortem C-U deamination 61, reflected in higher than expected percentage of Thymine in resulting sequences. We compared the percentages of nucleotides in sequences from our museum specimens and from live collected *N. lyristes*, which were almost identical (e.g., GC content 35.1% v. 36.2%). This indicates that sequence degradation has minor, if any, influence on our results.

In this study museum specimens replaced extant samples, necessitated by the rarity, or likely local extinction, of *N. lyristes* from most of its known range. Despite the additional challenges of working with museum specimens, aDNA can replace extant specimens when collection is either not possible because of extinction 61^,^^62^ or broad resampling is untenable 63^,^^64^.

With advances in the amplification of ancient DNA 65^,^^66^^,^^67^, museum collections are also opening up areas of study that are not possible with extant data alone 62^,^^67^^,^^68^. Ancient DNA can be utilized in the calibration of molecular clocks through dating tip ages 69. Samples from multiple time points, can provide additional information about the genetic and demographic changes in groups over time 70. Ancient DNA has been used in the reconstruction and timing of many mammal groups 52^,^^70^^,^^71^^,^^72^, but remains underutilized in the timing of insect lineages despite the abundance of specimens in museums. Several of the problems associated with the use of aDNA can be overcome by next generation sequencing (NGS). For example, NGS has the capability to target short and degraded DNA samples 73. NGS also allows for the sequencing of whole genomes from aDNA 64^,^^74^^,^^75^ and less destructive sampling techniques from Museum samples 76^,^^77^^,^^78^.

## Data Availability

All supplementary data are available at figshare: https://dx.doi.org/10.6084/m9.figshare.3100312.v2

Nucleotide sequences are available at GenBank: Accession numbers KU881748 - KU881763

## Competing Interests

The authors have declared that no competing interests exist.

## Corresponding Author

Gideon Ney: gideon.ney@gmail.com

## Editor

Due to Tree of Life Editor unavailability, a member of the *PLOS ONE* Editorial Board, Wolfgang Arthofer (University of Innsbruck, Austria), rendered the final decision on this paper.

## Appendix

**Supplemental Table 1: Table of primers designed for amplification of N. lyristes COI sequences:** Primers were designed from reference sequences of extant *N. lyristes*, *N. bivocatus*, and *N. robustus*.

Primer name	Primer sequence
lyF68 (forward)	5’-GGA ATT GCA CAT GCT GGA GC-3’
lyR197 (reverse)	5’-GTG ATA TTC CTG GGG CAC GT-3’
lyF187 (forward)	5’-ACG TGC CCC AGG AAT ATC AC-3’
lyR336 (reverse)	5’-CCG GCA GGA TCA AAG AAT GA-3’
lyF317 (forward)	5’-TCA TTC TTT GAT CCT GCC GGA-3’
lyR466 (reverse)	5’-GGC TTC CTT TTT CCC ACT TTC T-3’
lyF440 (forward)	5’-AGT CAA GAA AGT GGR AAA AAG GA-3’
lyR589 (reverse)	5’-AGC TGA AGT AAA ATA RGC TCG TG-3’
lyF545 (forward)	5’-ACA GTA GGA ATG GAT GTT GAT ACA C-3’
lyR694 (reverse)	5’-GCC TAG AGC TCA TAA AAG GGA AG-3’
lyF666 (forward)	5’-ACA GTC CTT CCC TTT TAT GAG CT-3’
lyR811 (reverse)	5’-AGA TAG AAC ATA ATG GAA ATG GGC T-3’

**Figure:**
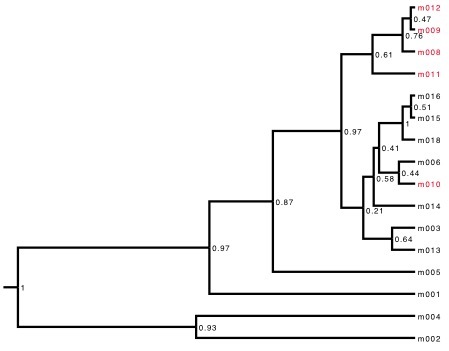
**Supplemental Fig. 1: Consensus tree using a strict molecular clock and the Midwest clade unconstrained.** Node values represent posterior probabilities calculated from eighteen million total trees. Red taxa represent Midwest samples and black taxa Atlantic samples.
